# Factors associated with SARS-CoV-2 infection and outcome in patients with solid tumors or hematological malignancies: a single-center study

**DOI:** 10.1007/s00520-021-06175-z

**Published:** 2021-04-14

**Authors:** Anouk Goudsmit, Edouard Cubilier, Anne-Pascale Meert, Philippe Aftimos, Konstantinos Stathopoulos, Chloe Spilleboudt, Angela Loizidou

**Affiliations:** 1 Internal Medicine, Institut Jules Bordet, rue Heger 1, 1000 Brussels, Belgium; 2 Clinical Trials Conduct Unit, Institut Jules Bordet, rue Heger 1, 1000 Brussels, Belgium; 3 Imaging Department, Institut Jules Bordet, rue Heger 1, 1000 Brussels, Belgium; 4 Hematology Department, Institut Jules Bordet, rue Heger 1, 1000 Brussels, Belgium

**Keywords:** SARS-CoV-2, COVID-19, Solid tumor, Hematological malignancies

## Abstract

**Background:**

Immunocompromised cancer patients are presumed to be at high risk of developing COVID-19 infection. Predisposing factors to contracting COVID-19 and to severe outcomes have been described in registries but were not compared between solid tumors and hematological malignancies.

**Method:**

This retrospective single oncologic center study included adults with solid tumors or hematological malignancies referred to testing by naso-pharyngeal swab for a SARS-CoV-2 RT-PCR from March 10 to May 18, 2020.

**Results:**

A total of 212 patients were included in the study. Forty-five (21%) were tested positive with SARS-CoV-2. The univariate analysis with positive SARS-CoV-2 PCR as a dependent variable reveals significant odds ratios (ORs) for age—with a mean of 62.5 years—(OR: 1.05, 95% CI: 1.02–1.08), performance status ≥2 (OR: 2.38, 95% CI: 1.22–4.70), inpatient status (OR: 2.36, 95%CI: 1.11–4.91), and hematological malignancies (OR: 2.48, 95% CI: 1.23–4.96). In contrast, OR for solid tumors reveals a negative association (OR: 0.40, 95% CI: 0.20–0.81). When integrating severe outcome (ICU admission or COVID-19-related death) as a dependent variable, the univariate logistic regression model shows significant ORs for pre-existing lymphopenia (OR: 4.0, 95% CI: 1.17–15.04), hematological malignancies (OR: 3.73, 95% CI: 1.09–13.80), and a negative association for solid tumors (OR: 0.27; 95% CI: 0.07–0.92).

**Conclusion:**

In patients referred for SARS-CoV-2 testing, hematological malignancies were associated with a higher risk of COVID-19 infection and severe outcomes. Other factors were age and inpatient status.

## Introduction

A novel coronavirus, named severe acute respiratory syndrome coronavirus 2 (SARS-CoV-2), was identified in China in December 2019 and caused a pandemic in a few weeks mostly by human-to-human transmission [1]. The infectious disease related to SARS-CoV-2 (COVID-19) produces various symptoms scaling from asymptomatic, to mild such as cough and fever, to severe such as acute respiratory distress syndrome (ARDS) and multiple organ failure (MOF) [2].

In the first studies, the diagnosis of COVID-19 was established by epidemiological and clinical suspicion, followed by thoracic computed tomography scanner (CT scan) in which the pattern evolves during the course of infection, starting with unilateral ground-glass opacification, to consolidation and bronchiectasis, to thickening of adjacent pleura and pleural effusion after 3 weeks, with a tendency to develop from a unilateral involvement at first, to a bilateral and multifocal form after a few days [3]. Genomic characterization of this new coronavirus [4] then allowed Corman et al. to develop the first real-time reverse transcription polymerized chain reaction (RT-PCR) for SARS-CoV-2 detection, to precise the diagnosis of COVID-19 [5], which has been used as diagnosis criteria in the more recent studies.

In cancer patients, community respiratory viruses are an important cause of respiratory infections [6], and adverse outcomes occur more frequently in comparison to immunocompetent patients [7]. In China, the prevalence of cancer in patients with COVID-19 was 2.0% which is higher than the overall cancer incidence of 0.29% [8]. Mortality rate in these patients is also increased in comparison with non-cancer patients [9, 10].

In the general population, risk factors for a more severe outcome of COVID-19 infection are older age, higher SOFA score, D-dimer > 1 μg/mL at admission [11], and co-morbidities [12].

Previous reports concerning cancer patients and COVID-19 highlighted the following predisposing factors for severe complications (ICU admission and death): Eastern Cooperative Oncology Group Performance Status (ECOG PS) ≥ 2 and progressive malignant disease [13], metastatic cancer patients [14], anti-cancer treatment administered within 14 days [15], hematological malignancies in comparison to health care providers with COVID-19 [16], and patients with solid tumors [17].

Our study aims to compare the factors associated with a SARS-CoV-2 infection and its outcomes among patients with solid tumors or hematological malignancies, as highlighted in a previously published study, but with the population of a tertiary oncological center in Brussels.

## Methods

### Study design

This study included all patients with hematological malignancies or solid tumors followed at Institute Jules Bordet who were referred for a nasopharyngeal swab between March 10, 2020, and May 18, 2020, during COVID-19 pandemic. Nasopharyngeal swabs were analyzed with the Altona RealStar® reverse transcriptase PCR SARS-CoV-2. Referral criteria were as follows: symptoms of COVID-19 (fever, cough, shortness of breath, desaturation, sore throat, rhinorrhea, headache, nausea, or diarrhea), a close contact with a confirmed case or incidental imaging findings compatible with COVID-19 infection (ground-glass opacity or crazy paving pattern). Patients with strong clinical or imaging signs of COVID-19 were tested twice if the first test was negative (by naso-pharyngeal swab or BAL). Onset time of the infection was based on the beginning of symptoms, or in asymptomatic patients the day of the naso-pharyngeal swab.

The study was approved by the Ethics Committee of the Institut Jules Bordet on May 26, 2020.

### Data collection

Collected data included demographics, symptoms, comorbidities, performance status, type and stage of cancer, anti-cancer treatment (chemotherapy, immunotherapy, or targeted therapy), blood work-up, CT scan, and severe outcome as defined by admission to the intensive care unit (ICU) or death attributed to COVID-19. Chronic steroid use was defined by a daily dose over 20 mg of prednisone per day with a cumulative dose over 700 mg [18].

### Outcome

The primary endpoint was the incidence of COVID-19 among patients with concordant symptoms, close contact with a confirmed case or concordant imaging. The secondary outcome was the occurrence of a severe outcome, consisting of an admission to the Intensive Care Unit (ICU) or death attributed to COVID-19 among positive patients.

### Statistical analyses

We used descriptive statistics to show the baseline demographic information of the included patients. A series of statistical tests were made for two different dependent variables, the positive SARS-CoV-2 RT-PCR and the severe outcome. The association between dependent variables and explicative variables was assessed by a chi-squared test with a threshold of significance defined by *p* <0.05, followed by a univariate analysis calibrated with 95% CIs (*p*-value < 0.05) for ORs. Finally, multivariate logistic analyses were carried out on significant variables at a 5% level in the univariate analysis.

## Results

### Characteristics of the included patients

A total of 212 patients were tested by RT-PCR for SARS-CoV-2: 163 for symptoms of COVID-19 (77%), 29 for incidental imaging findings compatible with COVID-19 (14%), and 20 for contact with a confirmed case (9%). Demographic, clinical, and tumor characteristics are described in Table 1. Mean age was 64 years with 55% female. Thirty-two (15.2%) were active smokers and 78 (37.0%) had a smoking history. A total of 155 (73%) patients had solid tumors and 57 (27%) hematological malignancies. The predominant anti-cancer treatment received was chemotherapy and concerned 94 (44.3%) patients, 82 (45.6%) had received anti-cancer treatments within 14 days, and 44 (24.4%) between 14 and 30 days from PCR-test day. Ninety-seven (46%) had a chronic lymphopenia (<1000/mm^3^) and 17 (8%) were on chronic steroid medication. Eighty-seven (41.0%) had a bad performance status of 2 or more and 44 (20.9%) were already hospitalized. Severity of lung damage is assessed with a score from 1 to 4 according to the classification of the Thoracic Imaging Society of the French Society of Radiology (http://www.sfrnet.org/rc/org/sfrnet/nws/News/2020/20200316-155630-175/src/nws_fullText/fr/CR TYPE COVID-19 LAST.pdf). A total of 169 (80%) chest CT-scans were performed with a global median pulmonary grade of 1 (Table 2).

**Table 1:** Characteristics of cancer patients with positive PCR SARS-CoV-2 Univariate analysis

***N = 45 (21%)***	**Ratio**	**OR**	**IC**	***p-value***
Age, median (years)	70	1.05	1.02-1.08	0.001
Sex, men (*n*, %)	23 (51%)	1.38	0.71-2.68	0.339
Solid cancer (*n*, %)	26 (58%)	0.40	0.20-0.81	0.01
Metastatic disease (*n*, %)	20 (77%)	2.48	0.98-7.15	0.069
Hematological malignancies (*n*, %)	19 (42%)	2.48	1.23-4.96	0.01
Remission	7 (15%)	2.38	0.84-6.34	0.088
Hypertension (*n*, %)	22 (49%)	1.43	0.73-2.77	0.291
Dyslipidemia (*n*, %)	12 (27%)	1.42	0.65-2.99	0.364
Diabetes (*n*, %)	8 (18%)	1.17	0.46-2.71	0.720
Stroke (*n*, %)	3 (7%)	1.01	0.22-3.42	0.985
Thromboembolic disease (*n*, %)	3 (7%)	0.72	0.16-2.32	0.622
COPD (*n*, %)	2 (4%)	0.32	0.05-1.16	0.137
Active smoker (*n*, %)	5 (11%)	0.64	0.21-1.65	0.396
Ex-smoker (*n*, %)	18 (40%)	1.18	0.59-2.30	0.635
BMI, mean kg/m^2^	25.7	1.03	0.96-1.10	0.433
Performance status > 2	15 (33%)	2.38	1.22-4.70	0.011
Inpatient (*n*, %)	15 (33%)	2.36	1.11-4.91	0.022
Chronic lymphopenia (n, %)	21 (47%)	0.98	0.50-1.89	0.946
Aplasia (*n*, %)	3 (7%)	2.30	0.46-9.76	0.267
Chemotherapy (*n*, %)	15 (33%)	0.56	0.27-1.10	0.097
Immunotherapy (*n*, %)	2 (4%)	0.39	0.06-1.41	0.212
Targeted therapy (*n*, %)	8 (18%)	0.91	0.37-2.07	0.833
Last treatment within 14 days (*n*, %)	17 (38%)	0.81	0.39-1.62	0.55
Last treatment from 14 to 30 days (*n*, %)	6 (13%)	0.46	0.16-1.10	0.102
Chronic steroid medication (*n*, %)	5 (11%)	1.61	0.49-4.63	0.393
CT chest (*n*, %)	38 (84%)			
Radiologic grade (SFR), median	2

**Table 2:** Univariate analysis for severe outcome of patients with cancer and positive PCR SARS CoV-2

***N = 18 (36%)***	**Ratio**	**OR**	**IC**	***p-value***
Age, median (years)	72	1.02	0.96-1.10	0.514
Sex, men (*n*, %)	10 (55%)	1.35	0.41-4.56	0.627
Solid cancer (*n*, %)	7 (39%)	0.27	0.07-0.92	0.04
Metastatic disease (*n*, %)	6 (33%)	2.14	0.26-45.74	0.525
Hematological malignancies (*n*, %)	11 (61%)	3.73	1.09-13.80	0.04
Remission	4 (22%)	2.29	0.44-13.07	0.322
Hypertension (*n*, %)	9 (50%)	1.08	0.32-3.59	0.903
Dyslipidemia (*n*, %)	5 (28%)	1.10	0.27-4.20	0.891
Diabetes (*n*, %)	4 (22%)	1.64	0.34-8.00	0.527
Stroke (*n*, %)	1 (5%)	0.74	0.03-8.27	0.808
Thromboembolic disease (*n*, %)	1 (5%)	0.74	0.03-8.27	0.808
COPD (*n*, %)	0			
Active smoker (*n*, %)	1 (5%)	0.34	0.02-2.55	0.351
Ex-smoker (*n*, %)	9 (50%)	2.00	0.59-6.97	0.266
BMI, mean kg/m^2^	26.4	1.06	0.92-1.25	0.435
Performance status > 2	2	1.26	0.38-4.36	0.712
Inpatient (*n*, %)	7 (39%)	1.51	0.42-5.39	0.520
Chronic lymphopenia (*n*, %)	12 (67%)	4.00	1.17-15.04	0.032
Aplasia (*n*, %)	1 (5%)	0.74	0.03-8.27	0.808
Chemotherapy (*n*, %)	7 (39%)	1.51	0.42-5.39	0.520
Immunotherapy (*n*, %)	1 (5%)	1.53	0.06-40.45	0.769
Targeted therapy (*n*, %)	5 (28%)	3.08	0.65-17.02	0.164
Last treatment within 14 days (*n*, %)	7 (39%)	0.98	0.27-3.47	0.975
Last treatment from 14 to 30 days (*n*, %)	4 (22%)	3.38	0.58-26.96	0.192
Chronic steroid medication (*n*, %)	3 (17%)	2.50	0.37-20.68	0.345
CT chest (*n*, %)	16 (89%)			
Radiologic grade (SFR), median	3			

### Characteristics and statistical analysis for positive SARS-CoV-2 PCR patients

Forty-five (21%) SARS-CoV-2 PCR were found to be positive: 33 among patients tested for symptoms of COVID-19 (20%), 3 for incidental imaging findings compatible with COVID-19 (10%), and 9 for contact with a confirmed case (45%). Demographic, clinical, and oncologic characteristics of this sub-group are described in Table 1. We found a mean age of 70 years in the positive and 62 years in the negative group. We included 51% of men in the whole population but we found a reverse sex ratio in the positive SARS-CoV-2 PCR group with 45% of men. The mean BMI was 25.7 in the positive group and 24.8 in the negative group. Distribution between solid and hematological malignancies in the positive group was reversed from the distribution in the whole population with 58% of solid tumor patients versus 42% of hematological patients (Table 3). The median performance status was 1 in the negative-PCR SARS-CoV-2 group and 2 in the positive group. Thirty-three percent were inpatients. Further examinations are described in Table 1. The median radiologic grade in positive tested patients was 2 versus 1 in negative patients, and 89% of patients who had a severe outcome presented a high median pulmonary grade of 3, compared to a median grade of 1 for the positive lab-test group with a non-severe outcome.

**Table 3:** Distribution of cancer patients

	All entrance			Clinical signs			Imaging			Contact tracing		
	Included patients	PCR SARS-CoV-2 positive	Severe outcome	Included patients	PCR SARS-CoV-2 positive	Severe outcome	Included patients	PCR SARS-CoV-2 positive	Severe outcome	Included patients	PCR SARS-CoV-2 positive	Severe outcome
All cancer	212	45 (21%)*	18 (40%)*	163 (77%)^¥^	33 (20%)*	15 (9%)*	29 (14%)^¥^	3 (10%)*	0	20 (9%)^¥^	9 (45%)*	1 (11%)*
Solid cancer	155	26 (17%)*	7 (27%)*	119 (77%)^¥^	19 (16%)*	7 (6%)*	28 (18%)^¥^	3 (11%)*	0	8 (5%)^¥^	4 (50%)*	0
Hematol cancer	57	19 (33%)*	11 (58%)*	44 (77%)^¥^	14 (32%)*	8 (18%)*	1 (2%)^¥^	0	0	12 (21%)^¥^	5 (42%)*	1 (20%)*

(%)* between PCR SARS-CoV-2 positive and included patients/between severe outcome and PCR SARS-CoV-2 positive

(%)^¥^ between clinical signs, imaging, contact tracing, and all entrance included patients

**Fig. 1:**
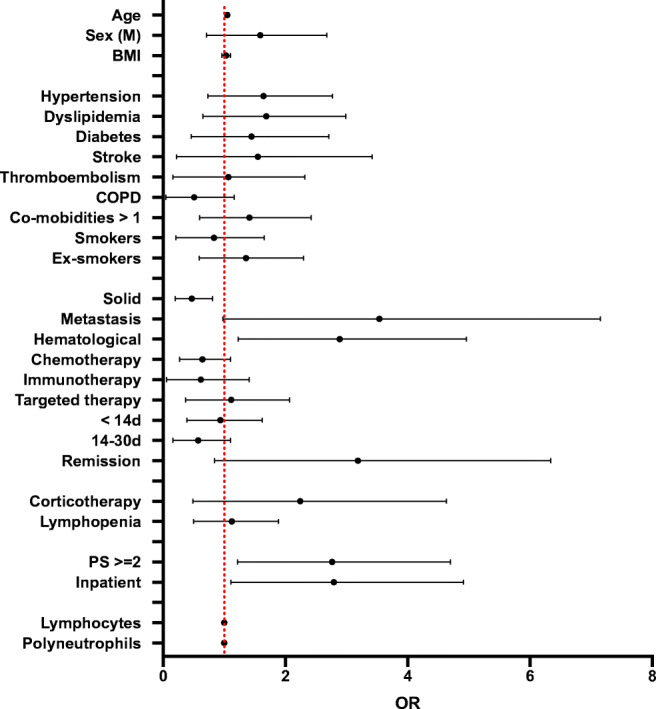
Univariate analysis for positive SARS-CoV-2 RT-PCR

A statistical analysis considering a positive SARS-CoV-2 RT-PCR as a dependent variable was performed. Chi-squared test results reveal significant association with age (*p*=0.001), ECOG performance status ≥2 (*p*=0.016), solid tumors (*p*=0.013), hematological malignancies (*p*=0.013), and inpatients (*p*=0.037). A univariate analysis shows significant positive ORs for a mean age of 62.5 years (OR: 1.05, 95% CI: 1.02–1.08), a ECOG PS ≥ 2 (OR: 2.38, 95% CI: 1.22–4.70), hematological malignancies (OR: 2.48, 95% CI: 1.23–4.96), and inpatients (OR: 2.36, 95% CI: 1.11–4.91). On the other hand, association between positive SARS-CoV-2 PCR and solid tumors appears negative (OR: 0.40, 95% CI: 0.20–0.81). The correspondent results are displayed in Table 1 and Fig. 1.

### Characteristics and statistical analysis for patients with severe outcome

Sixteen patients with positive SARS-CoV-2 PCR had severe outcome; 9 patients were admitted in the ICU and 7 died from COVID-19 symptoms. Among these patients, 15 (94%) were initially tested for compatible symptom with COVID-19 and only one patient for having a close contact with a confirmed case (Table 3).

**Fig. 2:**
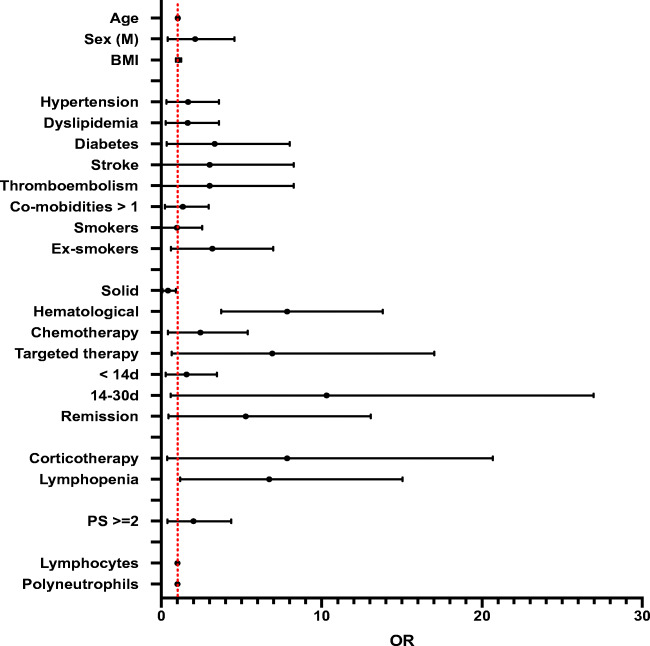
Univariate analysis for severe outcome

A statistical analysis was performed considering severe outcome in positive SARS-CoV-2 RT-PCR results as a dependent variable was performed (Table 2 and Fig. 2). We found a strong association for pre-existing lymphopenia (*p*=0.037) and a mean lymphocyte cell count of 881.5/mm^3^ at admission (*p*=0.018). A univariate analysis showed significant positive ORs for pre-existing lymphopenia (OR: 4.0, 95% CI: 1.17–15.04) and hematologic malignancies (OR: 3.73, 95% CI: 1.09–13.80), and a negative OR for solid tumors (OR: 0.27, 95% CI: 0.07–0.92).

## Discussion

Our results show that among cancer patients, patients with hematological malignancies have a higher occurrence risk of COVID-19 and of developing more severe outcomes. It also seems that symptomatic patients develop also worse outcomes than patients who were tested for other reasons.

As found in Kuderer’s cohort study, the most frequent solid cancer types was breast cancer [13], which is associated with a higher proportion of females included than males. We could not examine associations between the type of cancer and COVID-19 because of the low number of patients within each cancer type. In addition, we have not found any association between pulmonary metastasis and positive RT-PCR SARS-CoV-2 or severe outcome unlike in other studies [14].

We obtained an association with age coming out as a 5% risk increase to have a positive RT-PCR SARS-CoV-2 every each year, which correlates with results found in other publications [13].

Concerning medical history, we did not find any association between six chosen co-morbidities (hypertension, dyslipidemia, diabetes, thromboembolic disease, chronic obstructive pulmonary disease COPD) and a positive RT-PCR SARS-CoV-2 test or a severe outcome. This observation extends to cardiovascular co-morbidities, which, although being the most frequent co-morbidities in other studies that include non-cancer patients, had not shown significant statistical association with our dependent variables. This observation contradicts results found in other studies that included non-cancer patients [19] but mirrors those including cancer patients [9, 17]. This could be explained by the important impact of cancer itself on the studied variables.

We used the ECOG PS to split patients in two groups between good (PS < 2) and poor (PS ≥ 2) performance status. We found an association between the RT-PCR SARS-CoV-2 results and a bad PS in univariate logistic regression but also if the patient was hospitalized at the time of the swab, although these two co-variables are closely associated because patients are more hospitalized when their general condition is poor, and long hospitalizations tend to induce a lower PS. These results are similar to those recently published in the CCC19 cohort [13].

In contrary to other studies, we have not found any association between recent treatment, such as chemotherapy, targeted therapy, or immunotherapy, and COVID-19 or severe outcome. These results can be explained by a rapid application of oncology guidelines during COVID-19 pandemic (NICE, ESMO, EBMT…) which limited delivery of systemic anti-cancer treatments for stable cancer patients with COVID-19 (https://www.esmo.org/guidelines/cancer-patient-management-during-the-covid-19-pandemic. https://www.nice.org.uk/). It has been established that there can be a potential overlap between the coronavirus-related interstitial pneumonia and the possible pneumological toxicity of anti-PDL1 agents [20], but too few patients were included in this study to test this hypothesis. In the matter of treatments, we have not found any association between COVID-19 and chronic steroid use, which has not been highlighted in any other study either, despite known increased risk of infections in patients under such therapy at a cumulative level of 700 mg of prednisone [18].

At the beginning of the pandemic, there was limited available stock of personal protective equipment (PPE) and no PCR SARS-CoV-2 swab surveillance on admission day for hospitalized patients. We observed four nosocomial infections, one in the hematology unit, one in the palliative care unit, one in the oncology unit, and one in our surgical ICU. Moreover, 45% of patients who had contact with a confirmed case had a positive SARS-CoV-2 PCR (in comparison to 20% of positive PCR among patients with compatible symptoms and 10% among patients with compatible imaging). Thereafter, surveillance by nasopharyngeal swab was set up for every hospitalized patient and care-provider with at least one symptom suggestive of a COVID-19 nosocomial infection. It is important to note that two patients with a COVID-19 infection were in process of allogenic stem cell transplantation, one needed ICU care, and none of them died.

About the indication of having a nasopharyngeal swab test for COVID-19, 163 patients were tested for symptoms of COVID-19 (77%), 29 for incidental imaging findings compatible with COVID-19 (14%), and 20 for contact with a confirmed case (9%). During the same period, 426 patients visited the Emergency Department for all kind of reason, 627 patients were hospitalized (and so, at higher risk for having a contact with infected medical staff), and 1511 patients underwent a thoracic CT scan (including PET-CT). Among patients with positive RT-PCR for SARS-CoV-2, 45% of patients were initially tested for having a close contact with a confirmed case, in comparison to 20% of patients with compatible symptoms and 10% of patients with compatible imaging with COVID-19. However, 94% of patients with a severe outcome were initially tested because of compatible symptoms with COVID-19 and more precisely with respiratory symptoms (cough and desaturation) and/or fever. Among the 16 patients with a severe outcome, 7 were known for having a solid tumor (37% of solid tumor patients with a positive SARS-CoV-2 PCR) and 8 with a hematological malignancy (57% of hematological patients with a positive SARS-CoV-2 PCR).

About the correlation between CT scan and RT-PCR, a total of 197/212 patients underwent RT-PCR test and chest CT scan. 61/197 (31%) had negative RT-PCR test and negative CT findings. 72/197 (37%) had negative RT-PCR test and atypical CT findings for COVID-19 pneumonia. 46/197 (23%) presented positive RT-PCR test and positive CT findings for COVID-19. Nevertheless, our data does not allow to establish a statistical correlation between the positivity of the CT scan and the positivity of the RT-PCR. This question is explored, in the same population, in another study [21].

We highlighted an association with pre-existing lymphopenia and hematological malignancies, which are linked variables since in our population, 40% of patients with solid tumors had pre-existing lymphopenia against 60% in the hematological population.

When studying mortality by the severe outcome variable, we defined death related to COVID-19 by separating patients who presented worsening symptoms of COVID-19 (22%), from death not related to COVID-19 when from complications associated with their cancer (13%). In fact, with the nosocomial infection in our supportive care ward, and considering the difficulties of organizing outer hospital palliative care treatments for positive COVID-19 patients for sanitary reasons, end of life care occurred in the COVID-19 unit. Moreover, 93% of deceased patients, either positive or not for COVID-19, had active limitations of care.

The study has several limitations. First, it is a retrospective, single-center study. Second, the study took place during the first 3 months of the COVID-19 pandemic; as a consequence, we did not collect or analyze information about the clinical evolution of the patients, rehabilitation, and the post-COVID 19 syndrome beyond this limited study period. Another limitation is that, in the beginning of the pandemic, there was a lack of reagent for RT-PCR, so the nasopharyngeal swab was reserved only for patients with high suspicion of COVID-19 infection and undergoing anti-cancer therapy.

In conclusion, we confirm that among cancer patients, hematological malignancies have a higher occurrence risk of COVID-19 and develop more severe outcomes and that hygiene precautions must be introduced as soon as possible during a pandemic.

Further studies designed to explore the difference between solid and hematological patients with bigger sample sizes are needed to complete the available data on the impact of the intrinsic immunocompromised state and the immunosuppressive treatments on the seroprevalence, the seroconversion, the possibility of reinfection, the immunological protection after vaccination, and the survival after SARS-CoV-2 exposure.

## Data Availability

Data are extracted from the medical files of studied patients, after approval of the Ethical Committee of Institut Jules Bordet.

## Abbreviations

SARS-CoV-2: severe acute respiratory syndrome coronavirus 2
COVID-19: coronavirus infectious disease
RT-PCR: reverse transcription polymerized chain reaction
OR: odds ratio
95% CI: confidence interval at 95%
ARDS: acute respiratory distress syndrome
MOF: multiple organ failure
ECOG: Eastern Cooperative Oncology Group
PS: performance status
CT scan: computed tomography scan
ICU: intensive care unit
BMI: body mass index
COPD: chronic obstructive pulmonary disease
NICE: National Institute of Health and Care Excellence
PPE: personal protective equipment
